# Vessel Density and Retinal Thickness from Optical Coherence Tomography Angiography as New Indexes in Adolescent Myopia

**DOI:** 10.1155/2021/6069833

**Published:** 2021-12-15

**Authors:** Qin Zhu, Chunwen Chen, Jingyan Yao

**Affiliations:** Department of Ophthalmology, The First Affiliated Hospital of Soochow University, 188 Shizi Street, Suzhou 215006, China

## Abstract

**Purpose:**

To evaluate and quantify blood perfusion and retinal thickness (RT) from the perspective of quadrants by optical coherence tomography angiography (OCTA) in adolescents with myopia and explore the relationship between axial elongation and related indexes of OCTA.

**Methods:**

A total of 88 subjects (149 eyes) with different degrees of myopia were included in this cross-sectional study. Vessel density (VD) and RT of quadrants in macular and peripheral regions were measured through OCTA.

**Results:**

The superficial VD (SVD) of the parainferior region was significantly correlated with axial length (AL) between the emmetropia (EM) group and high myopia (HI) group (*P*=0.012). There were significant differences in deep VD (DVD) in all quadrants, except for the foveal, perifoveal, and peri-inferior regions (*P* > 0.05). However, there were significant alterations in the whole, parainferior, and perinasal regions (*P*=0.030, 0.023, and 0.035) in the low-to-moderate myopia (L–M) group compared with those in the HI group. There were significant differences in the RT in all quadrants, except for the foveal, paratemporal, and paranasal regions (*P* > 0.05) between the EM and L–M groups and the foveal region (*P* > 0.05) between the EM and HI groups. Nevertheless, only RT in the peri-inferior region of the L–M and HI groups showed significant differences. AL was negatively correlated with SVD in the perifoveal and parainferior regions (*r* = −0.179, *P*=0.029; *r* = −0.227, *P*=0.005) and inversely correlated with DVD and RT in almost all quadrants, except for the foveal region (*r* = −0.020, *P*=0.811; *r* = 0.135, *P*=1.000).

**Conclusion:**

DVD and RT were closely associated with the severity of myopia and might be new indexes in assessing and detecting myopia development via OCTA.

## 1. Introduction

Myopia is one of the main causes of visual impairment [1], which has generated serious economic and social consequences. Approximately 4758 million individuals will have myopia in 2050 worldwide [2]. The number of individuals with myopia in China has always been high and continues to be so [3]. The proportion of adolescents with myopia is increasing, which has attracted much attention from the society, especially the complications caused by excessive axial elongation, such as choroidal neovascularization, lacquer crack, myopic maculopathy, and glaucoma [4]. High metabolism in the retina is supported by a well-organized vascular system to obtain binocular vision function [5]. Dysregulated angiogenesis disrupts transportation of oxygen and nutrients, leading to unbalanced metabolic supply and retinal function abnormalities [6]. Great noninvasion and repeatability of optical coherence tomography angiography (OCTA) have been demonstrated in macular microvascular perfusion measurements [7]. OCTA allows quantifying visualization of retinal blood flow in patients with myopia. Evidence from previous studies had confirmed the retinal microvasculature abnormalities of high myopia through OCTA [8]. Considering that the retinal microvascular system is an important basis for maintaining visual function, it is essential that tiny structural changes at the early stage in myopic development should be carefully monitored to avoid pathophysiological outcomes of high myopia. To the best of our knowledge, few studies have been performed on adolescents by OCTA, so it is important to explore retinal changes in adolescents with myopia. This study described the quadrantal alterations of the retinal capillary and microstructure in adolescent myopia with a step-by-step pattern. It aimed to increase the understanding on the susceptibility of macular microcirculation and retinal thickness (RT) to axial elongation and explore the guiding role of these new indexes.

## 2. Materials and Methods

This was a cross-sectional study. All subjects and their guardians provided informed consent. This study was approved by the ethics committee of The First Affiliated Hospital of Soochow University. All recruitment and procedures strictly adhered to the principles of the Declaration of Helsinki.

### 2.1. Subjects

Subjects with all grades of myopia ranging from 0.75 D to −10 D (axial length (AL) ≥ 23.50 mm) who visited the Department of Ophthalmology of our institution from May 2020 to February 2021 were included in this project. Participants were categorized into the following three groups based on the AL: emmetropia (23.50 mm ≤ AL ≤ 24.5 mm), low and moderate myopia (24.5 mm < AL < 26 mm), and high myopia (AL ≥ 26 mm) [9]. All eyes had a best corrected visual acuity (BCVA) ≥ 16/20. The exclusion criteria were as follows: (1) intraocular pressure (IOP) ≥ 21 mmHg; (2) astigmatism of any eye < −1 D or > +1 D; (3) evidence of ocular disease other than myopia; (4) history of use of atropine at low concentration or wear of rigid gas-permeable lenses; (5) history of ocular trauma and ocular surgery; (6) presence of a systemic disease that might affect the blood flow, such as hypertension and diabetes mellitus; (7) age < 14 years or > 18 years; and (8) poor cooperation with quality of OCTA scan images <7/10.

### 2.2. Ophthalmic Examinations

All participants underwent comprehensive clinical ophthalmological examinations by an ophthalmic specialist, including BCVA, refractive status, slit-lamp biomicroscopy, funduscopic examination, tonometry by using a full auto tonometer TX-F (Topcon, Tokyo, Japan), and optical biometry using IOL Master (version 3.02, Carl Zeiss Meditec, Germany). All subjects underwent dilated optometry after two cycles of 5% compound tropicamide drops administration to determine the exact diopter.

### 2.3. OCTA

**Figure 1:**
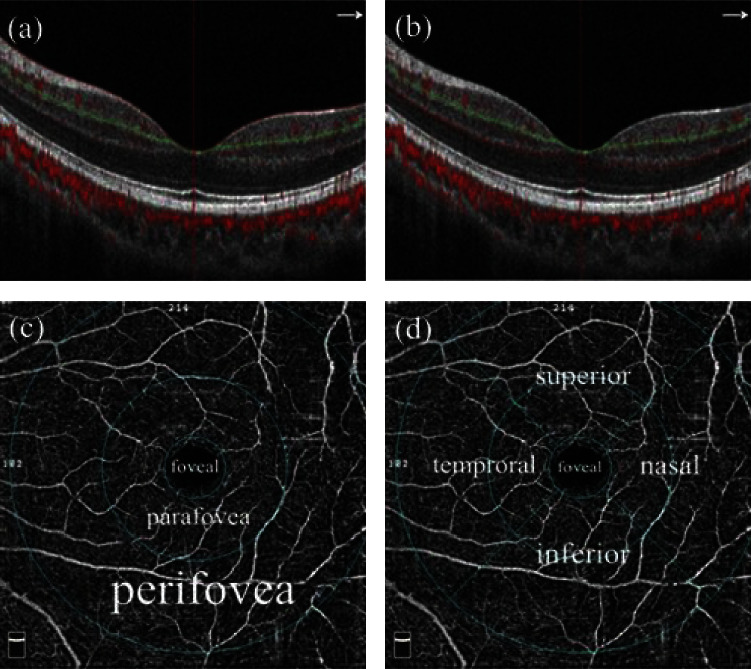
Pictorial representation of the macular capillary plexus. (a), (b) Superficial capillary plexus (SCP) and deep capillary plexus (DCP), respectively. (c) Three circles with different radii, including foveal regions, parafoveal regions, and perifoveal regions. (d) Superior, inferior, nasal, and temporal quadrants.

All patients were examined using OCTA (RTVue-XR, Optovue, Fremont, CA, USA) by one professional technician, and the information of subjects was concealed from the technician. The device frequency is set to 70 kHz, the wavelength is controlled at 840 mm, and the frequency width is set to 35 mm. The device was centered on the fovea for a 6 mm × 6 mm OCTA acquisition in the angioretina mode, and motion artifacts were removed, with the time controlled at approximately 2.9 s. Three-dimensional (3D) OCTA images were obtained by horizontal and vertical scans. A-scan area was centered on the fovea, and the B-scan was repeated at the fixed position [8]. RTVue software (version 2015.1.0.71) was used to segment the OCT scans and measure the figures in the regions of interest and divide microcirculation into the retinal superficial capillary plexus (SCP) and deep capillary plexus (DCP) to allow layer-by-layer visualization. The SCP is defined as the region between the internal limiting membrane and outer border of the ganglion cell layer. Respectively, the region between the outer border of the SCP and outer border of the outer plexiform layer is part of the DCP (Figure 1). Furthermore, images could be divided into three circles with different radii, which were measured in the following three regions: (1) the foveal region was defined as 1 mm radius centered on the foveal center; (2) the perifoveal region was defined as between 3 and 6 mm radius; and (3) the parafoveal region is located between the foveal and perifoveal regions. The parafoveal and perifoveal regions are further divided into four quadrants, namely, temporal, superior, nasal, and inferior [9]. Figures from the fovea and four quadrants in the parafovea and perifovea were finally analyzed to determine the differences among the three groups (Figure 1). To ensure the accuracy of the study, the images with quality <7/10 were eliminated. VD referred to the proportion of the total area occupied by the vessels, and RT was defined as the full layer.

### 2.4. Statistical Analysis

All data were analyzed using SPSS software (version 23.0; SPSS, Inc. Chicago, IL, USA). Qualitative variables are presented as numbers. Quantitative variables are presented as means and standard deviations. Qualitative variables were determined by the chi-square test. One-way analysis of variance was conducted to compare the differences among the three groups. Pearson's correlation was implemented to compare the AL and statistical significance of associations between variables. Only data for the eligible eye were used in the final analysis. A *P* value <0.05 was considered statistically significant.

## 3. Results

A total of 88 participants (149 eyes) were enrolled in the analysis. The demographic and ocular characteristics of all eligible subjects are presented in Table 1. The mean age was 15.97 ± 2.92 years (range, 14–18 years). There was no significant difference in age, sex, IOP, anterior chamber depth, and lens thickness among the three groups (all *P* > 0.05). Superficial vessel densities (SVDs) were 46.60% ± 0.56%, 45.54% ± 0.52%, and 45.91% ± 0.55% in emmetropia (EM), low-to-moderate myopia (L–M), and high myopia (HI) groups, respectively. Deep vessel densities (DVDs) were 50.12% ± 0.53%, 49.99% ± 1.00%, and 47.59% ± 0.41% in the three groups, respectively, and RTs were 301.25 ± 1.46 *μ*m, 294.06 ± 1.62 *μ*m, and 291.55 ± 1.84 *μ*m, respectively. The quadrant alterations of SVD in the L–M group were not statistically different from those in the EM group (Table 2). From the perspective of quadrants, DVD in the HI group was significantly lower than that in the EM group in most quadrants but in certain quadrants in the L–M group (Tables 3 and 4). The retina was remarkably thinner in the L–M group than in the EM group and especially in the HI group (Tables 5 and 4). However, independent of the segmentation, there was scarcely any statistically significant difference in RT between the L–M and HI groups, except for the peri-inferior region (Tables 5 and 4).

Generally, DVD and RT in most quadrants were associated with AL but not SVD. SVD in the perifoveal and peri-inferior regions was negatively associated with AL (*r* = −0.179, *P*=0.029; *r* = −0.227, *P*=0.005). DVDs in all quadrants were negatively correlated with AL, except for the foveal and peritemporal regions (all *P* < 0.05). The average RT in all quadrants were inversely correlated with AL, except for the fovea (all *P* < 0.05) (Table 4). Overall, SVD, DVD, and RT decreased following axial elongation, except in the fovea.

## 4. Discussion

In our study, parafoveal and perifoveal RT significantly decreased when emmetropia progressed toward low-to-moderate myopia, whereas only perifoveal inferior RT remarkably reduced when it progressed further toward high myopia. However, parafoveal and perifoveal RTs significantly thinned in the HI group compared with the EM group, and foveal RT thickened slightly with AL elongation. Interestingly, we also found the superior and inferior RTs in the parafovea changed—vertical changes—in early myopia and then the other quadrants changed significantly with AL extension in a step-by-step pattern. Our results are consistent with those of Wakitani et al. who demonstrated decreased peripheral RT, namely, parafoveal and perifoveal RTs in myopic eyes [10] and proposed that the absence of large blood vessels and optic fibers in the peripheral retina helps to withstand more stretching force for the entire retina to preserve the macular structure. Nevertheless, Li et al. reported that the retina generally becomes thin with AL elongation [11]. An essential reason is that their criteria for segmentation and grouping were evidently different from those of ours. Regardless of quadrantal changes, the retina becomes thin as it is pulled along the longitudinal growth of eyes.

In general, SVD in most quadrants remained unchanged in the process of axial growth. Notably, only the inferior of the perifovea remarkably decreased in the HI group compared with the EM group. Guo et al. also reported similar findings of SVD changes in the fovea; they found that not only the inferior but also nasal quadrants had lower SVD in high myopia [12]. Although only the perifoveal inferior of the HI group showed significant change, we speculated that the inferior quadrant possesses the earliest susceptibility to myopic development and is more prone to myopia complications based on the present study and that by Guo et al. [12]. At the same time, further long-term longitudinal studies are warranted. On the contrary, Golebiewska et al. reported a different finding that parafoveal SVD significantly decreased in myopia [13]. To our knowledge, the SCP is derived from the central retinal artery, the first branch of the ocular artery. A reasonable explanation is that the vascular endothelial cells maintain normal function via an intrinsic autoregulatory response to guarantee sufficient metabolic demands of the SCP [14]. However, the significant difference in the peri-inferior quadrant of SVD between the EM and HI groups could not be clearly explained.

As for the deep capillary, parafoveal and perifoveal DVDs showed no significant changes when emmetropia progressed toward low-to-moderate myopia, but it showed significant changes in high myopia. Through a deeper insight into quadrantal alteration, we found a remarkable reduction in all quadrants of the perifovea and the inferior, superior, and nasal quadrants of the parafovea in the comparison between EM and HI groups. In addition, the parainferior and perinasal quadrants considerably changed with further axial elongation, particularly when L–M progressed to HI myopia, which revealed a significantly negative correlation between the AL and nasal-inferior macular. Moreover, VD in the nasal quadrant was significantly correlated with the ocular perfusion pressure and retinal nerve fiber layer thickness [15], which corresponded with thinner nasal RT and may explain the high susceptibility of the nasal quadrant in our participants. Lee et al. demonstrated that VD measurements in the peripheral area were significantly reduced in highly myopic eyes [16]. Another study claimed that the peripheral DVD contributes as an indicator of capillary loss in high myopia [17]. However, Liu et al. found that the inferior and nasal quadrants were not correlated to AL and stated that the two quadrants had the lowest susceptibility during the development of myopia [18], which is contradictory to our findings, and the contrasting results may be ascribed to the difference in inclusion criteria and the population. Based on the abovementioned paradoxical results, subsequent studies should focus on the quadrant variation of microstructural and microvascular alteration with myopia progression. The theory of choroid ischemia and hypoxia has been illustrated in patients with myopia [19], and the thinning of blood vessels in some regions may be a response to blood flow redistribution [20]. The DCP contributed to partial oxygen supplementation to the inner segment of the photoreceptor [21]. In addition, it was the primary distinct region where venous drainage for the entire retinal capillary plexus exists [22], both indicating that further DVD decrease would result in retinal venous drainage disorder and retinal complications of myopia. The reason for the nonuniformity among the three circles is that the vessels were packed densely in the fovea and that the reduction was not significant compared with that in peripheral quadrants [23]. Although the fovea was not affected in our study, AL overextension would give rise to severe macular disease.

On the one hand, we inclined to the conjecture that the eyeball extended in the vertical direction of the perifovea in early myopia and then in all directions with further myopia development. On the other hand, we speculate that the susceptibility of RT to AL changes was higher than that of VD, and the VD of the deep capillary was higher than that of the superficial capillary, which may provide a new method to explore the myopic mechanism. Nevertheless, the confusion whether this retinal response precedes or follows the vascular compromise is still controversial [24]. Further longitudinal studies and specific mechanisms are required for additional investigation. Although the pathogenesis of choroid-associated myopia is not fully explained, current findings indicate that vascular insufficiency and RT changes may also be involved in the development of myopia, so we put forward the idea of VD and RT as new indexes in adolescent myopia and suggest the long-term monitoring role of VD and RT changes in myopia complications.

The primary limitations of this study lie in its small sample size and the single-center, cross-sectional design. Therefore, more multicenter, prospective studies with a large sample size should be conducted. Furthermore, the precise mechanism and function of decreased VD and thinner RT in myopia require comprehensive investigation; further studies focusing on the relationship between the microcapillary layer and myopia are warranted to supplement the results.

## 5. Conclusions

In conclusion, we utilized OCTA to assess and quantify the blood perfusion and RT of all degrees of myopia from quadrant perspectives. The information revealed some alterations in the retinal vasculature and microstructure, especially changes in RT in early myopia and VD alterations in high myopia. Besides, DVD and RT had greater susceptibility to AL than SVD. We speculated that DVD and RT might be related to myopia pathogenesis. Nonetheless, we have to emphasize the importance of VD and RT as new observational indexes in the diagnosis and treatment of myopia.

## Figures and Tables

**Table 1:** Demographic and ocular characteristics of participants between the three diagnostic groups.

Subjects	A	B	C	Average	*P* value
Age (years)	15.26 ± 3.02	16.11 ± 2.82	16.62 ± 2.81	15.96 ± 2.92	0.104
Gender (male/female)	23/32	25/29	19/21	66/83	0.892
IOP (mmHg)	15.44 ± 1.1	15.41 ± 1.09	15.88 ± 0.76	15.54 ± 1.03	0.057
AL (mm)	23.76 ± 0.68	25.33 ± 0.38	26.93 ± 1.13	25.18 ± 1.46	<0.01
ACD (mm)	3.81 ± 0.28	3.84 ± 0.36	3.88 ± 0.35	3.84 ± 0.33	0.65
LT (mm)	3.73 ± 0.22	3.83 ± 0.25	3.77 ± 0.21	3.77 ± 0.23	0.100

A, emmetropia, B; low-to-moderate myopia; C, high myopia, IOP, intraocular pressure; AL, axial length; ACD, amber chamber depth; LT, lens thickness; SE, spherical equivalent. Numbers appear as mean ± standard deviation or as median. Normally distributed data were analyzed by one-way ANOVA and nonnormal data analysis by the Kruskal–Wallis test. The chi-square test was applied to analyze the frequency of data for noncontinuous descriptive(s).

**Table 2:** SVD in the three diagnostic groups.

SVD/%	A	B	C	*P* value			
				A-B-C	A-B	A-C	B-C
Whole	46.60 ± 0.56	45.54 ± 0.52	45.91 ± 0.55	0.310	>0.05	>0.05	>0.05
Fovea	24.46 ± 9.20	28.09 ± 10.56	28.69 ± 1.53	0.506^*∗*^	>0.05	>0.05	>0.05
Parafovea	50.21 ± 4.37	49.15 ± 4.78	49.65 ± 4.28	0.367	>0.05	>0.05	>0.05
Paratemporal	50.35 ± 4.87	50.04 ± 4.86	50.40 ± 3.99	0.808	>0.05	>0.05	>0.05
Parasuperior	50.14 ± 4.97	49.39 ± 6.19	49.58 ± 4.42	0.859	>0.05	>0.05	>0.05
Paranasal	50.28 ± 5.23	49.32 ± 5.37	50.20 ± 3.79	0.55	>0.05	>0.05	>0.05
Parainferior	49.16 ± 5.64	48.34 ± 4.90	48.12 ± 4.99	0.579	>0.05	>0.05	>0.05
Perifovea	47.15 ± 5.81	45.30 ± 5.07	45.77 ± 5.02	0.077	>0.05	>0.05	>0.05
Peritemporal	45.79 ± 5.33	44.98 ± 4.42	44.35 ± 4.12	0.33^*∗*^	>0.05	>0.05	>0.05
Perisuperior	46.58 ± 6.44	44.81 ± 5.89	46.19 ± 5.80	0.229	>0.05	>0.05	>0.05
Perinasal	49.22 ± 7.28	47.32 ± 7.30	47.53 ± 6.85	0.265	>0.05	>0.05	>0.05
Peri-inferior	47.28 ± 6.85	44.24 ± 6.43	44.63 ± 6.98	***0.009***	0.072	***0.012***	1.000

A, emmetropia; B, low-to-moderate myopia; C, high myopia; SVD, superficial vessel density. Numbers appear as mean ± standard deviation for normally distributed variable. Normally distributed data were analyzed by one-way ANOVA and nonnormal data analysis by the Kruskal–Wallis test. ^*∗*^ANOVA was applied. *P* values that are significant are in bold and italicized.

**Table 3:** DVD in the three diagnostic groups.

DVD/%	A	B	C	*P* value			
				A-B-C	A-B	A-C	B-C
Whole	50.12 ± 0.53	49.99 ± 1.00	47.59 ± 0.41	***0.002***	0.926	***0.001***	***0.030***
Fovea	31.35 ± 9.63	30.98 ± 10.17	31.80 ± 10.89	0.928^*∗*^	>0.05	>0.05	>0.05
Parafovea	54.11 ± 3.56	53.14 ± 4.11	52.49 ± 3.62	0.112^*∗*^	>0.05	>0.05	>0.05
Paratemporal	54.29 ± 4.58	54.01 ± 4.09	53.31 ± 4.55	0.542	>0.05	>0.05	>0.05
Parasuperior	54.30 ± 3.62	53.50 ± 4.27	52.31 ± 3.34	***0.020***	0.813	***0.016***	0.230
Paranasal	54.77 ± 4.76	52.85 ± 5.64	50.32 ± 6.40	***0.001***	0.219	***0.001***	0.124
Parainferior	52.56 ± 4.75	52.29 ± 4.71	49.47 ± 4.88	***0.006***	1.000	***0.008***	***0.023***
Perifovea	50.10 ± 4.77	48.81 ± 5.62	47.17 ± 4.56	***0.007***	1.000	***0.006***	0.070
Peritemporal	50.16 ± 5.74	47.46 ± 6.24	46.34 ± 5.66	***0.021***	1.000	***0.018***	0.147
Perisuperior	50.06 ± 5.42	48.69 ± 6.19	47.33 ± 4.42	***0.017***	0.992	***0.014***	0.164
Perinasal	50.88 ± 5.83	50.44 ± 6.86	47.53 ± 6.01	***0.011***	1.000	***0.017***	***0.035***
Peri-inferior	48.75 ± 5.49	47.69 ± 6.24	45.40 ± 5.40	***0.009***	1.000	***0.009***	0.051

A, emmetropia; B, low-to-moderate myopia; C, high myopia, DVD, deep vessel density. Numbers appear as mean ± standard deviation for normally distributed variable. Normally distributed data were analyzed by one-way ANOVA and nonnormal data analysis by the Kruskal–Wallis test. ^∗^ANOVA was applied. *P* values that are significant are in bold and italicized.

**Table 4:** Correlation between AL and VD of superficial and deep and RT.

	SVD	DVD	RT
Whole	−0.120 (0.146)	−0.205 (***0.012***)	−0.389 (***<0.001***)
Fovea	0.156 (0.057)	−0.020 (0.811)	−0.135 (1.000)
Parafovea	−0.086 (0.297)	−0.227 (***0.005***)	−0.281 (***0.001***)
Paratemporal	−0.012 (0.884)	−0.199 (***0.015***)	−0.254 (***0.002***)
Parasuperior	−0.083 (0.314)	−0.213 (***0.009***)	−0.324 (***<0.001***)
Paranasal	−0.011 (0.895)	−0.300 (***<0.001***)	−0.232 (***0.004***)
Parainferior	−0.141 (0.085)	−0.197 (***0.016***)	−0.313 (***<0.001***)
Perifovea	−0.179 (***0.029***)	−0.229 (***0.005***)	−0.516 (***<0.001***)
Peritemporal	−0.155 (0.059)	−0.021 (0.803)	−0.500 (***<0.001***)
Perisuperior	−0.098 (0.233)	−0.184 (***0.025***)	−0.431 (***<0.001***)
Perinasal	−0.154 (0.060)	−0.294 (**<*0.001***)	−0.415 (**<0.001**)
Peri-inferior	−0.227 (***0.005***)	−0.271 (***0.001***)	−0.586 (**<0.001**)

AL, axial length; SVD, superficial vessel density; DVD, deep vessel density; RT, retinal thickness. Pearson's correlation was performed to test the relationship between AL with VD of superficial and deep and RT. *P* values that are significant are in bold and italicized.

**Table 5:** RT of full retina in the three diagnostic groups.

RT/*μ*m	A	B	C	*P* value			
				A-B-C	A-B	A-C	B-C
Whole	301.25 ± 1.46	294.06 ± 1.62	291.55 ± 1.84	***<0.01***	***0.009***	***<0.01***	0.769
Fovea	251.96 ± 11.90	253.43 ± 14.88	253.83 ± 13.70	0.454	>0.05	>0.05	>0.05
Parafovea	324.53 ± 12.53	317.32 ± 13.17	316.65 ± 11.93	***0.003*** ^*∗*^	***0.028***	***0.008***	1.000
Paratemporal	315.15 ± 13.24	309.30 ± 13.01	308.00 ± 11.42	***0.025***	0.152	***0.030***	1.000
Parasuperior	329.71 ± 12.57	323.24 ± 11.95	319.30 ± 12.26	***0.003***	***0.047***	***<0.01***	1.000
Paranasal	328.31 ± 12.71	322.70 ± 13.58	321.85 ± 12.51	***0.027*** ^*∗*^	0.133	***0.045***	1.000
Parainferior	324.44 ± 13.49	315.59 ± 15.99	314.78 ± 13.20	***<0.01***	***0.010***	***0.001***	1.000
Perifovea	289.49 ± 11.01	280.26 ± 13.92	274.18 ± 15.00	***<0.01***	***<0.01***	***0.005***	0.138
Peritemporal	274.82 ± 11.29	265.85 ± 13.85	260.38 ± 12.85	***<0.01***	***0.004***	***<0.01***	0.103
Perisuperior	292.73 ± 11.92	284.44 ± 14.72	279.28 ± 14.71	***<0.01*** ^*∗*^	***0.014***	**<*0.01***	0.178
Perinasal	310.09 ± 12.51	301.43 ± 16.23	297.43 ± 16.31	***0.001***	***0.028***	***0.001***	0.644
Peri-inferior	279.07 ± 12.01	269.50 ± 13.32	260.00 ± 18.43	***<0.01***	***0.005***	***<0.01***	***0.043***

A, emmetropia; B, low-to-moderate myopia; C, high myopia, RT, retinal thickness. Numbers appear as mean ± standard deviation for normally distributed variable. Normally distributed data were analyzed by one-way ANOVA and nonnormal data analysis by the Kruskal–Wallis test. ^∗^ANOVA was applied. *P* values that are significant are in bold and italicized.

## Data Availability

The original data, figures, and tables that were used to support the findings of this study are available from the corresponding author upon request.

## References

[1] Yekta A., Fotouhi A., Hashemi H. (2010). The prevalence of anisometropia, amblyopia and strabismus in schoolchildren of shiraz, Iran. *Strabismus*.

[2] Holden B. A., Fricke T. R., Wilson D. A. (2016). Global prevalence of myopia and high myopia and temporal trends from 2000 through 2050. *Ophthalmology*.

[3] Pan C.-W., Dirani M., Cheng C.-Y., Wong T.-Y., Saw S.-M. (2015). The age-specific prevalence of myopia in Asia. *Optometry and Vision Science: Official Publication of the American Academy of Optometry*.

[4] Saw S.-M., Gazzard G., Shih-Yen E. C., Chua W.-H. (2005). Myopia and associated pathological complications. *Ophthalmic and Physiological Optics*.

[5] Sun Y., Smith L. E. H. (2018). [WITHDRAWN] retinal vasculature in development and diseases. *Annual Review of Vision Science*.

[6] Potente M., Gerhardt H., Carmeliet P. (2011). Basic and therapeutic aspects of angiogenesis. *Cell*.

[7] Read S. A., Fuss J. A., Vincent S. J., Collins M. J., Alonso‐caneiro D. (2019). Choroidal changes in human myopia: insights from optical coherence tomography imaging. *Clinical and Experimental Optometry*.

[8] Min C. H., Al-Qattan H. M., Lee J. Y., Kim J.-G., Yoon Y. H., Kim Y. J. (2020). Macular microvasculature in high myopia without pathologic changes: an optical coherence tomography angiography study. *Korean Journal of Ophthalmology*.

[9] Khan M. H., Lam A. K. C., Armitage J. A., Hanna L., To C.-h., Gentle A. (2020). Impact of axial eye size on retinal microvasculature density in the macular region. *Journal of Clinical Medicine*.

[10] Wakitani Y., Sasoh M., Sugimoto M., Ito Y., Ido M., Uji Y. (2003). Macular thickness measurements in healthy subjects with different axial lengths using optical coherence tomography. *Retina*.

[11] Man R. E. K., Lamoureux E. L., Taouk Y. (2013). Axial length, retinal function, and oxygen consumption: a potential mechanism for a lower risk of diabetic retinopathy in longer eyes. *Investigative Opthalmology & Visual Science*.

[12] Guo Y., Sung M. S., Park S. W. (2019). Assessment of superficial retinal microvascular density in healthy myopia. *International Ophthalmology*.

[13] Gołębiewska J., Biała-Gosek K., Czeszyk A., Hautz W. (2019). Optical coherence tomography angiography of superficial retinal vessel density and foveal avascular zone in myopic children. *PLoS One*.

[14] Laties A. M. (1960). Central retinal artery innervation. *Archives of Ophthalmology*.

[15] Yun Y. I., Kim Y. W., Lim H. B. (2021). Peripapillary vessel parameters and mean ocular perfusion pressure in young healthy eyes: oct angiography study. *British Journal of Ophthalmology*.

[16] Lee M.-W., Kim J.-M., Shin Y.-I., Jo Y.-J., Kim J.-Y. (2019). Longitudinal changes in peripapillary retinal nerve fiber layer thickness in high myopia. *Ophthalmology*.

[17] Cheng D., Chen Q., Wu Y. (2019). Deep perifoveal vessel density as an indicator of capillary loss in high myopia. *Eye*.

[18] Liu M., Wang P., Hu X., Zhu C., Yuan Y., Ke B. (2020). Myopia-related stepwise and quadrant retinal microvascular alteration and its correlation with axial length. *Eye*.

[19] Al-Sheikh M., Phasukkijwatana N., Dolz-Marco R. (2017). Quantitative oct angiography of the retinal microvasculature and the choriocapillaris in myopic eyes. *Investigative Opthalmology & Visual Science*.

[20] Zhou M., Lu B., Zhang P., Zhao J., Wang Q., Sun X. (2017). Determination of topographic variations in inner retinal blood flow areas in young Chinese subjects using optical coherence tomography angiography. *Current Eye Research*.

[21] Scarinci F., Nesper P. L., Fawzi A. A. (2016). Deep retinal capillary nonperfusion is associated with photoreceptor disruption in diabetic macular ischemia. *American Journal of Ophthalmology*.

[22] Garrity S. T., Paques M., Gaudric A., Freund K. B., Sarraf D. (2017). Considerations in the understanding of venous outflow in the retinal capillary plexus. *Retina*.

[23] Hassan M., Sadiq M. A., Halim M. S. (2017). Evaluation of macular and peripapillary vessel flow density in eyes with no known pathology using optical coherence tomography angiography. *International Journal of Retina and Vitreous*.

[24] Wang X., Kong X., Jiang C., Li M., Yu J., Sun X. (2016). Is the peripapillary retinal perfusion related to myopia in healthy eyes? A prospective comparative study. *BMJ open*.

